# Secondary Prostate Lymphoma Mimicking Prostate Cancer Successfully Managed by Transurethral Resection to Relieve Urinary Retention

**DOI:** 10.3390/pathophysiology32030038

**Published:** 2025-08-02

**Authors:** Lorand-Tibor Reman, Ovidiu Malau, Daniel Porav-Hodade, Calin Chibelean, Arpad-Oliver Vida, Ciprian Todea, Veronica Ghirca, Alexandru Laslo, Raul-Dumitru Gherasim, Rares Vascul, Orsolya-Brigitta Katona, Raluca-Diana Hagău, Orsolya Martha

**Affiliations:** 1 Department of Urology, George Emil Palade University of Medicine, Pharmacy, Science, and Technology of Targu Mures, 540139 Targu Mures, Romania; tibor.reman@umfst.ro (L.-T.R.); calin.chibelean@umfst.ro (C.C.); arpad.vida@umfst.ro (A.-O.V.); ciprian.todea@umfst.ro (C.T.); maria.ghirca@umfst.ro (V.G.); alexandru.laslo@umfst.ro (A.L.); raul-dumitru.gherasim@umfst.ro (R.-D.G.); orsolya.martha@umfst.ro (O.M.); 2 Clinic of Urology, County Clinic Hospital of Târgu-Mureș, 540139 Targu Mures, Romania; ovidiu_malau@yahoo.com (O.M.); raresvascul@gmail.com (R.V.); 3 Doctoral School of Medicine and Pharmacy, George Emil Palade University of Medicine, Pharmacy, Science and Technology of Targu Mures, 540139 Targu Mures, Romania; 4 Department of Anaesthesiology and Intensive Care, County Emergency Clinic Hospital of Târgu-Mureș, 540139 Targu Mures, Romania; katona.brigics@gmail.com; 5 Department of Pathology, County Clinic Hospital of Târgu-Mureș, 540139 Targu Mures, Romania; ralucahagau1995@gmail.com

**Keywords:** prostate lymphoma, mantle cell lymphoma, secondary prostate lymphoma

## Abstract

Secondary lymphoma of the prostate is described as the involvement of the prostate gland by lymphomatous spread from a primary site. This condition is exceedingly rare and often presents diagnostic and therapeutic challenges. The symptoms often mimic those of benign prostatic hyperplasia or prostate cancer, including LUTS (lower urinary tract symptoms) and even complete urinary retention. Here, we present a rare case of a 62-year-old male patient undergoing chemotherapy for stage IV mantle cell stomach lymphoma and subsequently secondary prostatic involvement. The patient presented with complete urinary retention, accompanied by biochemical (PSA = 11.7 ng/mL) and imaging (Magnetic Resonance Imaging-PIRADS V lesion) suspicion for prostate cancer. Histopathologic analysis of the MRI-targeted prostate fusion biopsy revealed secondary prostatic lymphoma. The chosen treatment was transurethral resection of the prostate (TUR-P) for relief of symptoms, which significantly improved urinary function (postoperative IPSS = 5 and Qmax = 17 mL/s). This case underscores the importance of considering prostatic lymphoma in the differential diagnosis of bladder outlet obstruction, especially in patients with a known lymphoma history. This report also provides a focused review of the literature on secondary prostatic lymphoma, highlighting the diagnostic challenges, treatment options, and clinical outcomes.

## 1. Introduction

Acinar adenocarcinomas are the most common malignancy types of the prostate, representing the vast majority of prostate tumors [1]. In contrast, primary or secondary lymphomas of the prostate are exceedingly rare, accounting for only a tiny fraction of prostatic neoplasms. According to the 2022 World Health Organization (WHO) classification, prostate tumors are divided into two main categories: epithelial neoplasms of the prostate and mesenchymal tumors unique to the prostate. Epithelial tumors are further classified into glandular neoplasms of the prostate and (baso)squamous neoplasms. Glandular cancers include prostatic cystadenoma, high-grade prostatic intraepithelial neoplasia, intraductal carcinoma, prostatic acinar adenocarcinoma, prostatic ductal adenocarcinoma, and treatment-related neuroendocrine prostatic carcinoma. The (baso)squamous prostate cancer group comprises adenosquamous carcinoma, squamous cell carcinoma, and adenoid cystic carcinoma types. Unique mesenchymal tumors of the prostate are categorized as stromal tumors and are subdivided into two types: prostatic stromal cancers of uncertain malignant potential and prostatic stromal sarcoma. Based on this classification, lymphoid neoplasms of the prostate are classified separately from unusual epithelial tumors, reflecting their uncommon occurrence [2].

Secondary prostate tumors are rare, with most cases identified incidentally at autopsy. The majority of secondary prostatic malignancies are the result of direct invasion by bladder cancer, which should be distinguished from distant metastasis. The most common primary sites of secondary prostate cancer are lung cancer, pancreatic carcinoma, and melanoma, while tumors originating from the gastrointestinal tract, kidney, endocrine organs, and germ cells are less frequent [3].

Mantle cell lymphoma is an uncommon subtype of B-cell non-Hodgkin lymphoma, characterized by a t(11;14) genetic translocation involving the CCND1 gene (cyclin D1). Unfortunately, by the time of diagnosis, the disease is frequently widespread. Common signs and symptoms of mantle cell lymphoma are the so-called “B symptoms,” which include swollen lymph nodes, fevers and chills, night sweats, unintended weight loss, and abnormal blood counts, especially pancytopenias, resulting from bone marrow infiltration. Extranodal involvement is also common, particularly in the gastrointestinal tract, spleen, and bone marrow [4]. This rare and aggressive lymphoma accounts for approximately 5% of all B-cell non-Hodgkin’s lymphomas [5], with a 3:1 male-to-female ratio [6]. Primary and secondary lymphomas of the prostate are exceedingly rare [7,8] with only a limited number of publications addressing this pathology [9]. Prostatic mantle cell lymphoma is even less common, with only 11 reported cases [10].

Based on its rarity, prostatic lymphoma can be a diagnostic challenge. In men with a history of lymphoma presenting with obstructive uropathy or other urinary symptoms, prostatic lymphoma should be considered in the differential diagnosis [11]. Diagnosis is typically based on prostate biopsy, transurethral resection, or prostatectomy specimens (while assessing for benign prostate hyperplasia or prostate cancer).

## 2. Detailed Case Presentation

We present the case of a 62-year-old man with an established history of mantle cell non-Hodgkin lymphoma, accompanied by bladder outlet obstruction and complete urinary retention. He reported several weeks of lower urinary tract symptoms, especially voiding difficulties (weak stream, hesitancy, and nocturia), which led to complete urinary retention, necessitating emergency catheterization. On digital rectal examination, the prostate gland was found to be enlarged and seemed benign.

The patient’s medical history was significant for hematological disease. In 2019, a biopsy of a laterocervical adenopathy, along with coxal medullary aspirate and staging imaging (four-region computed tomography), confirmed stage IIIA mantle cell non-Hodgkin lymphoma, with immunohistochemistry demonstrating a mantle cell phenotype (CD20+, CD5+, Cyclin D1+, BCL2+). The patient’s ECOG (Eastern Cooperative Oncology Group) performance status grade was 0. The patient’s Mantle Cell Lymphoma International Prognostic Index (MIPI) score was 3.51 points (age = 59 years, ECOG performance status = 0, white blood cell count = 12 × 10^9^/L, LDH = 220 U/L), indicating an intermediate-risk category with a median overall survival of 51 months. He was treated with eight cycles of R-CHOP chemotherapy (rituximab plus cyclophosphamide, doxorubicin, vincristine, and prednisone). In June 2023, he presented relapsed symptomatology with gastrointestinal involvement, and stage IVA mantle cell non-Hodgkin lymphoma was detected based on stomach and colonic biopsies, leading to treatment with a Bruton-kinase inhibitor (ibrutinib).

**Figure 1:**
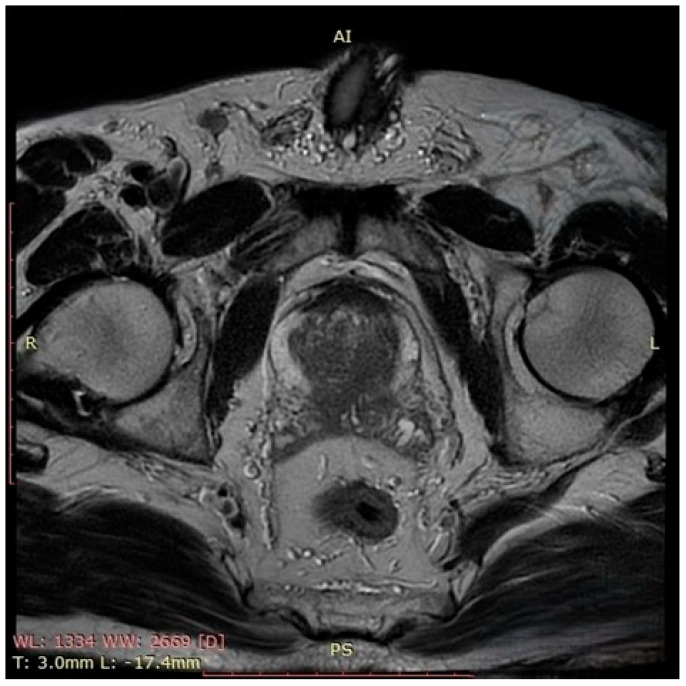
This axial T2-weighted image of the prostate demonstrates a hypointense lesion located in the peripheral zone, bilaterally, which is ill-defined, with disruption of the normal high-signal intensity of the peripheral zone and focal capsular bulging. There is no clear evidence of extracapsular extension or seminal vesicle invasion. These findings are suggestive of a PIRADS 5 lesion and highly suspicious for clinically significant prostate cancer.

In October 2023, the patient presented with complete urinary retention and an indwelling urethrovesical catheter in place. A thoraco-abdomino-pelvic staging CT scan identified two pulmonary nodules and multiple cervico-thoraco-abdomino-pelvic lymph nodes without any sign of active hematological disease. A PET-CT scan performed at that time showed multiple bilateral latero-cervical lymph nodes, without evidence of significant diffuse 18F-FDG uptake. Unfortunately, despite our efforts, we were unable to retrieve the original imaging files or the formal radiology report. The patient exhibited no systemic symptoms such as night sweats, fatigue, fever, or weight loss and remained active. His medication regimen included ibrutinib for non-Hodgkin lymphoma, Perindopril for grade I hypertension, Omeprazole as a gastroprotective agent, and Allopurinol for high uric acid levels. The patient was a non-smoker and non-drinker without any family history of autoimmune disorders or cancers, including prostate cancer. The patient’s ECOG performance status grade was also 0, but the MIPI score was 3.47 points, still indicating an intermediate-risk category with a median overall survival of approximately 51 months. Given the patient’s clinical–biological status and considering the risks and limitation of prostate cancer screening, the decision was to measure the PSA level. Following two consecutive measurements of the Prostate-Specific Antigen level that showed elevated levels (11.7 ng/mL), multiparametric prostate Magnetic Resonance Imaging (MRI) (Figure 1) was performed. The prostate MRI demonstrated a bilateral ill-defined lesion in the peripheral zone, with Prostate Imaging Reporting and Data System (PIRADS) 5 characteristics, highly suggestive of malignancy. Considering the laboratory and imaging investigations, MRI fusion-guided transrectal prostate biopsy was indicated and performed. The histopathological findings described secondary prostatic lymphoma.

A multidisciplinary tumor board was conducted, which involved both oncologists and urologists. Given the patient’s urinary retention and long-term catheterization, the relief of obstruction was a priority. The decision was made to proceed with surgical debulking of the prostate to restore urinary flow.

The preoperative blood work showed a white blood cell count of 12.230/mm^3^ (normal: 3.790–10.330/mm^3^), consisting of 1.770/mm^3^ neutrophils (normal: 1.500–7.700/mm^3^), 9.240/mm^3^ lymphocytes (normal: 1.100–4.000/mm^3^), and 820/mm^3^ monocytes (normal: 100–900/mm^3^). The hemoglobin was 14.4 g/dL (normal: 13.9–17.7 g/dL), hematocrit was 42.1% (normal: 39.6–51.8%), and platelet count was 367.000/mm^3^ (normal: 150.000–370.000/mm^3^). The creatinine concentration was 1.01 mg/dL (normal: 0.6–1.1 mg/dL) and lactate dehydrogenase concentration was 134 U/L (normal: 100–250 U/L). The blood coagulation profile, serum electrolytes, and liver function tests were within the normal ranges.

The patient underwent a transurethral resection of the prostate (TUR-P). During surgery, a 4 cm long prostate with bilobar enlargement and a median lobe was observed. The length of the surgery was 60 min, removing approximately 25 g of tissue. There were no intraoperative complications, and a 22Fr three-way urinary catheter was inserted. The patient’s postoperative recovery was uneventful. He could void spontaneously without residual volume after catheter removal on postoperative day 4. At the first postoperative follow-up, his urinary symptoms had markedly improved: his International Prostate Symptom Score (IPSS) was 5, and maximum urinary flow rate (Qmax) was 17 mL/s, as measured using uroflowmetry.

**Figure 2:**
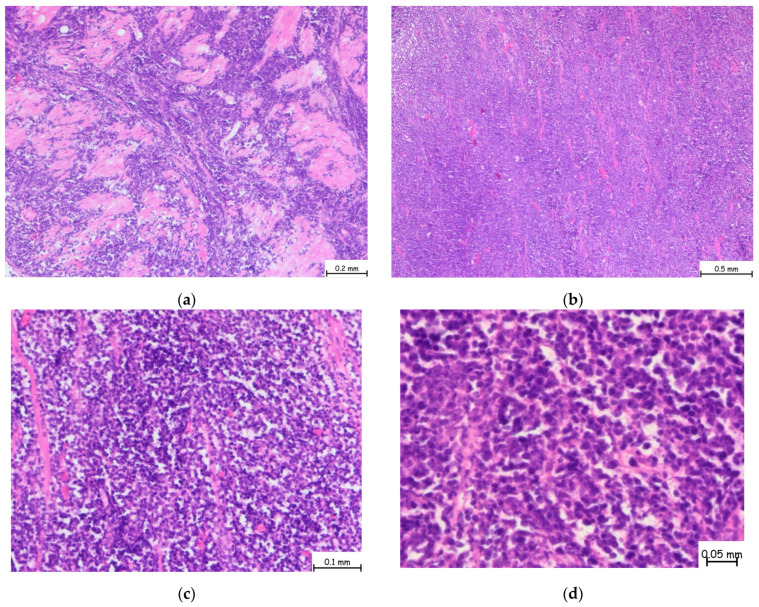
Hematoxylin and Eosin staining of prostate tissue showing diffuse lymphoid infiltration replacing nearly the entire glandular architecture at microscopic magnification of (**a**) 5×, (**b**) 10×, (**c**) 20×, (**d**) 40×. The infiltrate is predominantly composed of small- to medium-sized atypical lymphoid cells with irregular nuclear contours and scant cytoplasm. The normal prostatic glands are significantly reduced. These morphologic features are consistent with a dense, neoplastic lymphoid infiltrate, suggestive of lymphoproliferative prostate disorder.

**Figure 3:**
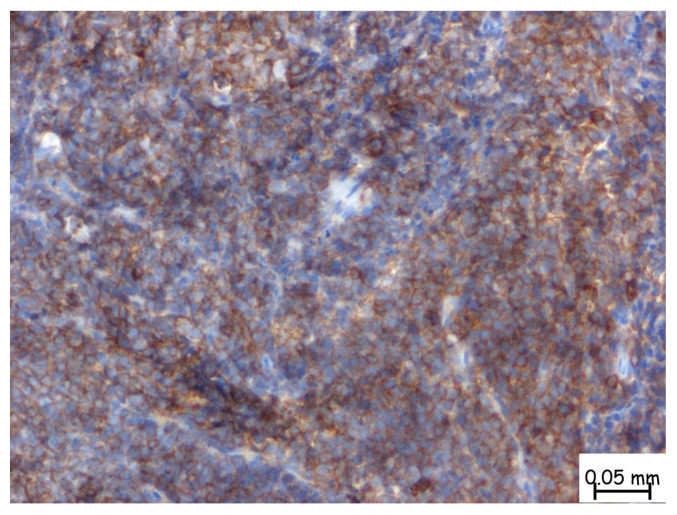
A dense infiltrate of small- to medium-sized lymphoid cells within the prostatic stroma. These cells demonstrate strong, diffuse membranous positivity for CD5, seen as a brown chromogenic reaction. The immunoreactivity is widespread and uniform across the neoplastic population, indicating homogeneous expression of CD5 among the infiltrating lymphoid cells. The microscopic magnification is 40×.

**Figure 4:**
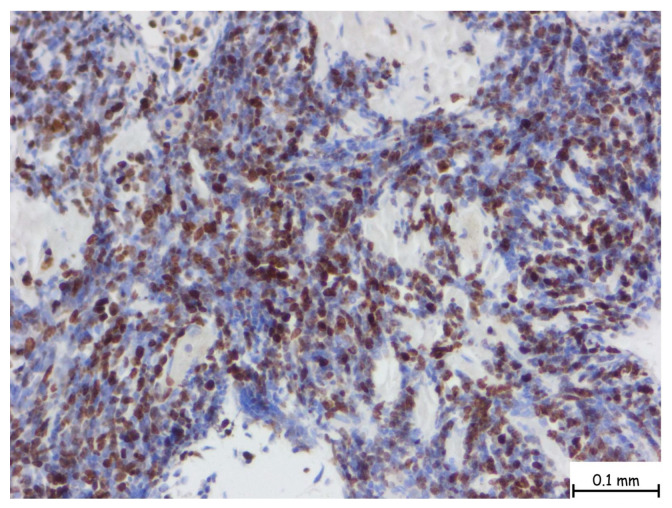
The nuclear brown staining pattern is characteristic for Ki-67, a well-established marker of cellular proliferation. In this case, numerous nuclei are positive for Ki-67, indicating a significant fraction of actively cycling cells. The Ki-67 proliferation index is intermediate to high, with an estimated labeling index ranging from 30% to 60% depending on region, suggestive of a more aggressive biological behavior or a higher-grade lymphoma subtype. The microscopic magnification is 40×.

The microscopic examination revealed a lymphoproliferative process characterized by a diffuse lymphoid infiltrate, with focal areas displaying a vaguely nodular structure. The infiltrate consisted of medium-sized cells, among which, large cells with slightly irregular nuclei and no prominent nucleoli were consistently observed. This process involved nearly all examined tissue fragments and disrupted the glandular architecture of the prostatic parenchyma (Figure 2a–d). Immunohistochemical staining with CD20 confirmed a mature B-cell origin (Figure 3). The Ki67 proliferation index was 60–70%. Given the patient’s prior histopathological diagnosis, additional immunohistochemical tests were performed to confirm mantle cell lymphoma, including CD20, CD3, Ki67, Bcl-2, Bcl-6, MUM1, Cyclin D1, CD23, CD5, and CD10. The tumor cells exhibited strong and diffuse expression of Bcl-2, CD5, and Cyclin D1, while CD23, Bcl-6, MUM1, and CD10 were not expressed. This immunophenotype is consistent with mantle cell lymphoma (Figure 4).

After surgery, the patient was referred back to the hematology department to continue the management of systemic lymphoma.

We consulted our hematology colleagues regarding the patient’s case. Unfortunately, after the diagnosis of secondary prostatic involvement from mantle cell lymphoma, the patient’s overall condition progressively worsened while still receiving ibrutinib. By January 2025, his ECOG performance status had declined to 3, considerably restricting further therapeutic interventions. Given the patient’s declining functional status and inadequate response to treatment, the hematology team decided to pursue best supportive care.

## 3. Discussion

Here, we presented the case of a patient with a history of gastric lymphoma undergoing chemotherapy, who later presented with complete urinary retention and was diagnosed with secondary prostatic lymphoma through prostate biopsy. Prostatic lymphoma, whether primary or secondary, often presents with non-specific urinary symptoms. Our patient’s obstructive voiding symptoms and elevated PSA level, combined with suggestive prostate MRI results, initially pointed toward a diagnosis of prostate adenocarcinoma, illustrating the diagnostic challenge. This underscores the importance of a correct diagnosis. A high degree of suspicion based on the patient’s history of lymphoproliferative disease and a confirmatory biopsy are crucial whenever clinical findings are atypical.

Mantle cell lymphoma represents between 4 and 9% of all lymphoma types [10]. This type of lymphoma is characterized by an aggressive clinical course with many relapses after traditional chemotherapy, which requires a complex therapeutic protocol [12]. In a report by the American Cancer Society published in 1998, which compared 62 cases of malignant lymphomas involving the prostate, no significant difference was found in median survival between primary and secondary prostatic lymphomas. In their study, the mean age at diagnosis was 62 years, and six patients were younger than 40 years old [13]. In our case, the patient was 62 years old at diagnosis, with a history of four years of chemotherapy. Mantle cell lymphoma is a mature B-cell neoplasm that primarily affects elderly patients, with a clear male predominance. Borrelia burgdoferi infection has been proposed as a moderate risk factor for this pathology. Variations in the interleukin-10 and tumor necrosis factor genes plus a family history of hematologic malignancies have all been proposed as a potential risk factors for developing mantle cell lymphoma [14]. This distinct type of lymphoid neoplasm has an average survival time of three years. More recent population-based data suggest improvements in overall survival with modern therapies. The multivariate analysis found significant correlations between age, performance status, and presence of B-symptoms with overall survival [15]. Our patient’s course reflects the relapsing nature of mantle cell lymphoma: he initially responded to R-CHOP chemotherapy but the disease progressed a few years later, requiring second-line treatment. Fortunately, at the time the prostate became involved, his performance status was excellent, and he had no systemic B-symptoms, which are favorable factors prognostically.

Prostatic lymphoma is rare, with only around 200 reported cases, most of which are discovered post-mortem [16]. Mantle cell prostate lymphoma is even rarer, with only 11 cases reported in the literature [10]. Patients typically present with progressive lower urinary tract symptoms. Treatment generally involves chemotherapy or radiation, with surgery reserved for mechanical obstruction. Classification of prostatic lymphoma as primary or secondary depends on its location and the absence of extraprostatic disease. According to the criteria published by Bostwick and his colleagues, primary prostatic lymphoma is diagnosed when the primary symptoms are attributed to prostatic enlargement, and lymph nodes, the liver, or the spleen are not involved within one month of diagnosis, with the major bulk of disease localized to the prostate [13]. In the presented case, the patient clearly had secondary prostatic lymphoma given his known disseminated MCL.

In 2003, Chim et al. [17] published the first case of prostatic mantle cell lymphoma case in a 73-year-old patient who had a 6-month history of lower urinary tract symptoms, such as urgency, frequency, hesitancy, and poor urine stream, and also presented with acute urinary retention. At physical examination, generalized enlarged lymphadenopathies were detected. They performed both a groin lymph node biopsy and transrectal prostatic biopsy, which showed similar lymphomatous infiltrates. After six months of chemotherapy (a combination of cyclophosphamide, vincristine, procarbazine, and prednisolone), the patient achieved complete remission [17]. Since 2003, only ten more cases have been published in the literature. PSA level elevation was noted in four cases, three of which were associated with underlying prostate cancer, while only one patient had elevated PSA without prostate cancer [10]. Elevated PSA levels are uncommon in prostatic lymphoma; the most common presenting symptoms are lower urinary tract symptoms, especially urine retention [18]. Notably, our patient had a PSA level of 11.7 ng/mL, which is considerably elevated, representing an uncommon finding in prostatic lymphoma. The review of published cases revealed that significant PSA level elevation generally only occurs in those with concurrent prostatic adenocarcinoma [10].

Once diagnosed, the management of prostatic lymphoma should be tailored to the individual patient’s disease extent and symptoms. Given that lymphoma is a generalized disease, systemic therapy is the mainstay of treatment for almost all patients. This treatment typically involves a combination between chemotherapy and immunotherapy [19]. Surgical interventions, such as TUR-P or HoLEP, are generally reserved for managing complications of local tumor mass effects, such as urinary tract obstruction [20]. In our case, TUR-P was performed solely to relieve bladder outlet obstruction. It is important to emphasize that surgery in these cases does not treat the lymphoma itself but rather addresses the mechanical issue.

We conducted a literature review to identify previously reported cases of mantle cell lymphoma involving the prostate, either as a primary or secondary lymphoma (Table 1).

Overall, the prognosis of prostatic MCL is influenced by the lymphoma’s characteristics and stage. While local control of lower urinary tract symptoms can be improved with interventions like TUR-P, long-term outcomes depend on the response to systemic therapy.

## 4. Conclusions

Physicians should consider the possibility of malignant lymphoid involvement of the prostate when encountering dense lymphoid infiltrate on a microscopic examination for lower urinary tract obstruction. Additionally, prostate lymphoma should be highly suspected in patients with previously diagnosed systemic lymphoma. An accurate diagnosis is critical, as management of this type of cancer differs greatly from that of typical prostate cancer.

An important aspect of patients with hematological diseases is the screening for urological diseases, such as lower urinary tract obstruction. Early identification of urinary tract obstruction allows for timely intervention, enhancing the overall quality of life. Integrating routine urologic assessments into the care of hematology patients should be a priority for healthcare providers to ensure comprehensive and multidisciplinary disease management. Close collaboration between urologists, oncologists, and pathologists is essential for comprehensive care that addresses both the local and systemic aspects of their disease.

## Figures and Tables

**Table 1:** Summary of published cases on primary and secondary prostatic lymphomas.

	Year	Age	Primary/Secondary	Symptom(s)	Diagnostic Method	Treatment	PSA	Associated Cancer
Chim CS [17]	2003	73	Secondary	Acute urinary retention	Prostate biopsy	Chemotherapy	-	None
Chu PG [8]	2005	65/80	Secondary/Secondary	No documented LUTS	Prostate biopsy TUR-P	No documented TUR-P	- -	None None
Abdussalam A [7]	2013	82	Secondary	Bladder outlet obstruction	TUR-P	TUR-P	2.4	None
Coyne JD [11]	2012	60	Secondary	Prostatism + recurrent urinary tract infection	Prostate biopsy	Not documented	-	None
Chen B [21]	2012	83	Primary	LUTS, urinary retention	TUR-P	TUR-P	3.2 ng/mL	Bladder cancer
Gurioli A [9]	2013	83	Primary	Gross hematuria + weight loss	Transvesical adenomectomy	Transvesical adenomectomy	-	Renal cancer
Rajput AB [22]	2014	74	Secondary	Elevated PSA levels	Prostate biopsy	Chemotherapy	17.16 ng/mL	Prostate adenocarcinoma
Petkovic I [23]	2016	64	Secondary	Fatigue, splenomegaly, elevated PSA level	Prostate biopsy	Chemotherapy + Androgen Deprivation Therapy	52 ng/mL	Prostate adenocarcinoma
Milburn PA [12]	2017	59	Secondary	Progressive LUTS	HoLEP	HoLEP	1.2 ng/mL	None
Tvrdíková E [24]	2019	64	Secondary	Elevated PSA level	Radical prostatectomy	Radical prostatectomy	5.9 ng/mL	Prostate cancer
Ünal S [10]	2021	70	Primary	LUTS + elevated PSA level	Prostate biopsy	Chemotherapy	8.2 ng/mL	None

Note: LUTS = lower urinary tract symptoms; TUR-P = transurethral resection of the prostate; PSA = Prostate-Specific Antigen; HoLEP = Holmium Laser Enucleation of the Prostate.

## Data Availability

The original contributions presented in this study are included in the article. Further inquiries can be directed to the corresponding author.
